# Supplementary material for: Patterns of Intron Gain and Loss in Fungi
Source: PLoS Biol. 2004 Nov 30;2(12):e422. doi: 10.1371/journal.pbio.0020422 (PMC532390; doi:10.1371/journal.pbio.0020422)
Supplement: Table S1 — Also available at http://genes.mit.edu/NielsenEtAl/. (4.3 MB ZIP). [file pbio.0020422.st001.zip › NielsenEtAl/html/311.html]

AN7014.1.NCU07866.1.MG06395.1.FG05423.1


```
 CLUSTAL W (1.82) Multiple Sequence Alignments - Introns Inserted


Sequence 1: MG06395.1	1445 aa
Sequence 2: FG05423.1	1436 aa
Sequence 3: NCU07866.1	1431 aa
Sequence 4: AN7014.1	1162 aa
Alignment Length: 1462 aa
Number Identitical Residues: 557 aa
Alignment Score (without introns) 31721


MG06395.1 	MPTAKRQKGARKSENNEGSSTHPPSTATNQRGGESE-----FSQLAHQHWLKPSKRT--T
NCU07866.1	MPPAKRVKSSASADKGPAAGSGRPTTQDLE--GDSE-----FANLARQHWLKATKRADDV
FG05423.1 	MPTVKRHKGSGAAKAQKQSDPKPKPTDGRPTPAEVEEEEHQFVQLARKHWLKSGKKP--A
AN7014.1  	------------------------------------------------------------
          	                                                            

MG06395.1 	KVKIKNDVLKS-MWDSLERDQFPHKSLLELETLQLLES2YLWPGYTENSSHHHVLLTVLI
NCU07866.1	KVKVKNDVLKSEIWDALEKDGFPLKSLLVLEGLQTLER~HVSPSPKR-------------
FG05423.1 	KPKVKNDVLKQNVWDVLEREGFQYKPLLLLESLQTLES2YLWPGYTEEASNFHVLLIALI
AN7014.1  	--------------------------------------~---------------------
          	                                                            

MG06395.1 	V~NTKRRERLDTWA~LFEDRPVDFSSLFRRVLSMSIDQTLSIATRTQIMCFVIQGFQSLD
NCU07866.1	F1NAKRRERLDAWG1LFNDRPADFSDLFRRALSMTLDDSLSWSIRTQVLQFIIYAFESLD
FG05423.1 	T~NVKHREHLATWS1LFEDRPADFSSLFRRLLSMMIDRTLSVTLRTQLLCFFIYAFQSLD
AN7014.1  	-~------------~---------------------------------------------
          	                                                            

MG06395.1 	CGIVRKECAPLVSISIWHNLVTEEKRDHLLDQDLHLHKAWRAALRRYEAADDATKARLRF
NCU07866.1	CAIVRKECAPLVSISIWHNLSTEEKRNELLDSNPQLRKTWRASAKRYDAADDAGKARLRF
FG05423.1 	CTLVRKECAPLVSIGIWHNLSTEQSREASLEQLPHLRKAWKAAHKRYEAADEQNKARLRF
AN7014.1  	---------------------------------------------------------MRF
          	                                                         :**

MG06395.1 	ERSWLYTLVLDFYSLLYGGNSTPA1DVVIYCERFMELLIDLQSQLPTRRYVNTLLQDLHI
NCU07866.1	ERAWLYSSVLEFFRVLYTENAK-Q~DQVMYCERFLEFLIDVLSQLPTRRYANTLLQDLHI
FG05423.1 	ERSWLYTLLLDFLGLLYTQNSK-A~DQVLYCERFTEFLTDLQSQLPTRRYVNALVQDLHI
AN7014.1  	ERSWLYTMLLDFLRRLNGAEKDQA~DNLRYCERFLEFLVDLESQLPTRRYVNTLLKDLHL
          	**:***: :*:*   *   :     * : ***** *:* *: ********.*:*::***:

MG06395.1 	STAITISPLYNDEDHGLFRDLHGLFQHFTFFIINDYTGAQLSSAEAYGRKCAELAKLQRI
NCU07866.1	LPALRLSPMYNDEGNGLLRDLAKLLSHFVYFTVDDQTGVQYSKTEAYDQHCARLAKLQRT
FG05423.1 	IPAARLSPMFNDEENTLLRDLQTLLSHFTFFDINDQTGAQYSIKEAYDKHCESLAKLQRT
AN7014.1  	LPLMRLSRLYRSADNALFRDFHNLLKHFTGFAINDYTGETLSTQAMYDAHCHDLARLQRT
          	 .   :* ::..  : *:**:  *:.**. * ::* **   *    *. :*  **:*** 

MG06395.1 	AFQHFREKLLVLALSNYGSIGDRGELVTLLEPLTDDEVSRLASLLLLRTSYPDAAKLPVD
NCU07866.1	SLKHFKDKLTVLALSNYGSIDKRSDLFGLLDVLTDEELTELARLLGLRISYPESSTLVVD
FG05423.1 	ALKHFREKLTVLALSNYGAVDQRQELEASLQPLTDEELMELVSLLGFRTEYPDSLSLPVD
AN7014.1  	SMKHFKDKLTILALSNYGSIEQRSELEGQLKALDDSELQDLCAHLGFRTSYPKQANVAAD
          	:::**::** :*******:: .* :*   *. * *.*:  *   * :* .**.  .: .*

MG06395.1 	RKFLIEVLLSAFEKRQTFRDVIRDMNIMPTEQTLFESSLLRTDSYNGSQPLALPKLNLQY
NCU07866.1	RRFLMEVLLSTFERHKTFQEEAAELSVLPTEETLFETGLRRTDHYDGSRPLALPKVNLQY
FG05423.1 	RKLLLEVVLSNFERRKTFQEAARHMSLAPTEETLFDSSFQQAESYDGSHPMALPKLNLQY
AN7014.1  	RHLYMEVLLSYHERTTSFQEATSNLDVVPTEESLYDPSLLRNETYDGSRPLAIPKLNLQY
          	*:: :**:** .*:  :*::   .:.: ***::*::..: : : *:**:*:*:**:****

MG06395.1 	LSIGDFLWRAFVLYRCESFYGIRKDVEAALQRLKPISPRPGETS-FTGNSRMALPVSKPA
NCU07866.1	LSVGDFLWRSLTLYRCEAFYAIRQDIEAALARLKPEARRTGETV-FTGFSRMALPISKPT
FG05423.1 	LSVGDFLWRALVLYRCESFYGVRKDIETAIRRLRPESKRSDETN-FAGFSKMAMPISKPA
AN7014.1  	LSLGDFLWRSFLLYRAEAFFQIRKDMELIVKRMQPRSSQDGRTLTFDGFSRMAIPIPKPA
          	**:******:: ***.*:*: :*:*:*  : *::* : : ..* :* * *:**:*:.**:

MG06395.1 	2VVDVVPAQVGDDAPSLVKAEVTIDLRRLTDGVKRDWDSLRPDDVVFLLAVDASTSQENE
NCU07866.1	~ILDVVPPLVGTDVPSTVKAEVTIDLRRLTEHVRRDWDALRPDDVLFLVEVNASKAKNVE
FG05423.1 	~ILEVAPPLVGDDKPSTVRAEVSFDVRRLGEGVSREWDSLRQDDVVFLIAVEPSPTKSAS
AN7014.1  	~IIEVAPPKVGSTKPAFVRAEITIEVGRLADHVRTEWDSLRPDDVVFLLAVQPG--NQGK
          	 :::*.*. **   *: *:**::::: ** : *  :**:** ***:**: *:..  :. .

MG06395.1 	TGGPPLGEAQKQGLLAVRTAEVMQIVDDRGRAVRD-------DGFGRGAIRRLILKLDPN
NCU07866.1	NGGAPLSEAERLGLVTVRTAEVIQIMDDRGRAVRDAQA--YFENHNRSYARKIQVRLDAH
FG05423.1 	NGEENLSESERLGVITVRTAEIHQITDDKGRQVRD-------GAQSLDSKRRIQLKLDPH
AN7014.1  	YGFRDVEAPQTPGIVHVRSADIVQVLDENGRPLREPASGQTNGYRSRPRVRRLLVNLDSA
          	 *   :  .:  *:: **:*:: *: *:.** :*:. :..     .    *:: :.**. 

MG06395.1 	MYKADIEGPSSAKSDIYQRFNVVVRRSGRENNFKPVLESIRSLALTDVPLAPWLHEVFLG
NCU07866.1	AYKRDSE----GKRNVYDGINLIVRRSGRENNFKPVLESIRDLTLSEVPLAPWMHDVFLG
FG05423.1 	AYSRDAERAAAGKPDVYGKINLLLRRGRRENNFKPVLESIRNLVLSDVPLPEWLHEVFLG
AN7014.1  	AFKADKDRTSQGKPDIYPLINVVARRKARENNFKSILETMQRLIASDITLPQWIQDIFLG
          	 :. * : .: .* ::*  :*:: **  ******.:**::: *  :::.*. *::::***

MG06395.1 	YGDPAAATFKLLPNRIKTLDYRDTFLDWQHLVASLPGRIVEPGFDVDGSFGPPYVLTTAD
NCU07866.1	YGDPAGATYKNLPNRIKKLDYRDTFLDWQHLIESLPGKNVEPSEDVSGTINPPFVLETVE
FG05423.1 	YGDPAGAYYKNLPNRERKVDFRDTFLDWQHLAESLPGKIIDPGDDVSGSFGPPYVLESVE
AN7014.1  	YGDPAGACYTELPNRLKSVDFRDTFLDWQHLIESFPGLTIEPSGDATSSFQPPYVLEYVE
          	*****.* :. **** :.:*:**********  *:**  ::*. *. .:: **:**  .:

MG06395.1 	KPEEEVSTKQSKKRRRDAEPALISEVETLKVSTYKTPNNGPYPTDRLKRNNVRFTPAQID
NCU07866.1	KPAEDGASKPSKKRRRDAEPALISEIETVKVSSYKPPNNGPYPIDAPKLNSVRFTPKQVE
FG05423.1 	KQSEPPSTKPSKKRRRDADPALIAEIETLKVSSYKPPNNGPYPIDNPKVNPVRFTPAQIE
AN7014.1  	ESAQPSTSSAPKKRRRDQHAEERGGPKSLRVSTYKPPNPGPYPVDAPRLNAVRFTPAQIQ
          	:  :  ::. .****** ..   .  ::::**:**.** **** *  : * ***** *::

MG06395.1 	AIISGSQPGLTVIVGPPGTGKTDVATQIINNIYHNFPEQRTLLIAHSNQALNQLFTKIVA
NCU07866.1	AIISGSQPGLTVVVGPPGTGKTDVATQIINNIYHNFPEQKTLLIAHSNQALNQLFAKIVA
FG05423.1 	AITSGTQPGLTVIVGPPGTGKTDVATQVINNIYHNHPEQKTLLLAHSNQALNQLFAKIVA
AN7014.1  	AIASGTQPGLTVIVGPPGTGKTDVATQIINNIYHNFPTERTLLVAHSNQALNQLFQKIVA
          	** **:******:**************:*******.* ::***:*********** ****

MG06395.1 	LDIDERHLLRLGHGEEDLDAGGNFGKAGRVESFLKNRDRYLLEVTKLAVSIGAPGAHGNS
NCU07866.1	LDIDERHLLRLGHGEEDLETEGSFSKHGRVESFLENRDRYLLEVNRLAASIGAPGAHGNS
FG05423.1 	LDIDERHLLRLGHGEEDLGTEGNFSKHGRVESFLENRDRYLLEVRKLATSLGAPGAHENS
AN7014.1  	LDIDERHLLRLGHGEEELDTETSY------------------------------------
          	****************:* :  .:                                    

MG06395.1 	AETAGHFNSV~YVEPAWARFLAFTDDPSLSAAEVAAAFPFTDFFADAPQPLFPPEGDREA
NCU07866.1	AETAGYFNKV~YVQPAWAKFSELTKAEDATAEDIVKAFPFHYYFADAPQPLFPPDADVDT
FG05423.1 	AETAGYFNNV~YVVPAWNRFQLVASDDASTVSDIMEAFPFHAYFADAPQPIFPPEGDKEQ
AN7014.1  	---------I1YIRPAWTKFWELARSENTSTEEIIAAFPFHEYFSNAPAPVFDPSASKET
          	         : *: *** :*  .:     :. ::  ****  :*::** *:* *... : 

MG06395.1 	VSDIINGCYRHISRIFAELADILPFEILRREKDKANYLLTSEARIVAMTSTHAAIRRGEI
NCU07866.1	VREIANGCYRHISKIFTELADVMPFEILRRDRDKENYLLTNEARIIAMTATHAAMRRGKI
FG05423.1 	AIEVAKGCYRHISKIFLELADALPFEILRRDRDKANYLLTSEARIIAMTTTHAAIRRGEI
AN7014.1  	VVDVAEGCQRHIDRIFSELEDIRPFEILRQPKDKANYLLVKEARIIAMTSTHAAMRRQEI
          	. :: :** ***.:** ** *  ******: :** ****..****:***:****:** :*

MG06395.1 	ASLGFKYDNVVVEEAAQITEIESFIPLAMQKPKAGHNGLQRIVLCGDHLQNSPVIQNMAF
NCU07866.1	ASLGFHYDNVVMEEAAQVTEIENFIPFAMQKPKDGKVPLQRIVLCGDHYQNSPVIQSLAF
FG05423.1 	ASLGFQYDNVVMEEAAQVTEIETFLPLAMQKPRNGKMGLQRVVLCGDHFQNSPVIQSLAF
AN7014.1  	ADLGFHYDNIVMEEAAQITEVESFIPTALQNMKEGQLPLKRIVLCGDHLQNSPIIQNLAF
          	*.***:***:*:*****:**:*.*:* *:*: : *:  *:*:****** ****:**.:**

MG06395.1 	RHYANLEQSLFSRLVRLGVPTISLDQQGRARPSIAKLYQWRYNNLGNLPHVETSDEFLTA
NCU07866.1	RHYANLEQSLFSRLVRLGVPTINLDLQGRARPSISSLYKWRYPSLNDLPHTMSATEFLTA
FG05423.1 	RHYANLEQSLFSRLVRLGVPTVALDQQGRARGSIASLYQWRYPKLDNLPSVQTSPEFVKA
AN7014.1  	RQYAHFEQSLFLRLIRLGVPAITLDQQGRARPSIAELFRWRYQNLGNLPIVEQAPEFKQA
          	*:**::***** **:*****:: ** ***** **:.*::*** .*.:** .  : **  *

MG06395.1 	NAGFKYDYQFINVPDYKGKGESEPSPHFIQNLGEAEYAVAIYQYMRLLGYPANKISILTT
NCU07866.1	NAGFRYDYQFIDVPDYKGQGESEPTPHFIQNLGEAEYAVAIFMYMRLLGYPAEKISILAT
FG05423.1 	NAGFKYDYQFINVPDYKGQGEAEPTPHFIQNLGEAEYAVAIFQYMRLLGYPAEKITILTT
AN7014.1  	NAGFQFEYQFINVPDYQGTGEREPTPHFVQNLGEAEYAVAIFQYMRLLGYPASKISILAT
          	****:::****:****:* ** **:***:************: *********.**:**:*

MG06395.1 	YAGQRALVRDVLKHRCGRGQIFGLPKIVTTVDKYQGEQNDY1IILSLTRTARVGYLRDVR
NCU07866.1	YAGQRALIKDVLGHRCAKNPIFGMPRIVTTVDKYQGEQNDY1IILSLTRTSRVGYLRDIR
FG05423.1 	YAGQRALVKDVLSHRCARNPVFGLPKAVATVDKYQGEQNDY1IILSLTRTSRVGYLRDVR
AN7014.1  	YAGQTALIKDVLNHRCSKNTLFGMPKIVTTVDRYQGEQNDY1IILSLTRTRTVGYLRDVR
          	**** **::*** ***.:. :**:*: *:***:******** ********  ******:*

MG06395.1 	RLTVALSRARLGLYVVGRRQVFESCPELRQAFDILLQRPDKLSLVTGELWPSQRSLADET
NCU07866.1	RLTVALSRARLGLYILGRRSVFESCYELREAFELLLQRPDKLTLVTGELWPSQRVLADEV
FG05423.1 	RMTVALSRARLGLYILGRREVFEACPELHPAFDLLLQRPDKLMLVTGELWPTPREVTEEP
AN7014.1  	RLTVALSRARLGLYILGRREVFESCYELKPAFDLLLQRPDKLMLAPGEMYPATRTLDDEV
          	*:************::***.***:* **: **::******** *..**::*: * : :* 

MG06395.1 	DKETVPGEVVMEDVVHMGQYVYEMTVTKMGQIGT-GKGLPMLEEVEEEAAGAVEALAPPP
NCU07866.1	GKEGLEGETVMEGVEHLGQYVFEMTKTRIEQLKEEGKQLPAPGESEAQVEVMAAEGEEGA
FG05423.1 	G--AVEGEVPMEGVEHLGQYVFEMTKTRIKQLQE-EQGEPIIEEEFEDGEGEYGNGD---
AN7014.1  	KG------TPMEGVEHLGQYVFEMTQAKIKALGG--EGAIVDETMPDEEGDLIEDGDE--
          	        . **.* *:****:*** :::  :    :          :            

MG06395.1 	AASELMEVTEDREVEGIDPEIEDAGVAAN
NCU07866.1	EVVELAQ-PDEEEEEAVRVEGFEAEEE--
FG05423.1 	--------DGEDDEDDIEADILEVREGE-
AN7014.1  	---EMLGGGEEGDEDPLHEHVYA------
          	   .      : : : :  .
```
